# 131. A Pharmacist Led Antimicrobial Stewardship Pilot at Discharge Improves Outpatient Antibiotic Utilization

**DOI:** 10.1093/ofid/ofab466.333

**Published:** 2021-12-04

**Authors:** Kayla Hiryak, Geena Kludjian, Jason C Gallagher, Jason C Gallagher, Marissa Cavaretta

**Affiliations:** 1 Temple University School of Pharmacy, Philadelphia, Pennsylvania; 2 Temple University, Philadelphia, PA

## Abstract

**Background:**

The impact of antimicrobial stewardship programs has been well observed in institutional settings; however, patients complete over one-third of their antibiotic course after discharge. This creates a gap in stewardship efforts at transitions of care. We studied whether pharmacist review of antibiotic prescriptions at discharge would improve outpatient antibiotic prescribing.

**Methods:**

This was a pilot project of patients in medicine wards of an academic medical center who were discharged on oral antibiotics between February and May 2021. Patients who were pregnant, <18 YO, had COVID-19, or leaving against medical advice were excluded from evaluation. For the pilot, a verification queue was created in the electronic health record (EHR) system where orders for discharge antibiotics were reviewed by investigator pharmacists before prescriptions were electronically sent to outpatient pharmacies. During the pilot, prescriptions were reviewed Monday-Friday afternoons from 12pm-4pm. Data was collected on incidence, type, and acceptance rate of pharmacist interventions, and a cost savings analysis was conducted with values calculated by the EHR system.

**Results:**

There were 149 patients included with oral antibiotic prescriptions reviewed during the time frame. Of those patients, 48 (32.2%) had at least one prescription that was intervened on by a pharmacist. A total of 55 interventions were made with an acceptance rate of 76%. The median time for pharmacist review was 10 minutes (IQR 5-15). Patients who received infectious diseases (ID) consultation during admission required less intervention than patients without expert consultation but did not reach significance (8/35 and 47/114 respectively, p=0.07). The total cost savings associated with all interventions was &20,743.00.

Table 1. Interventions

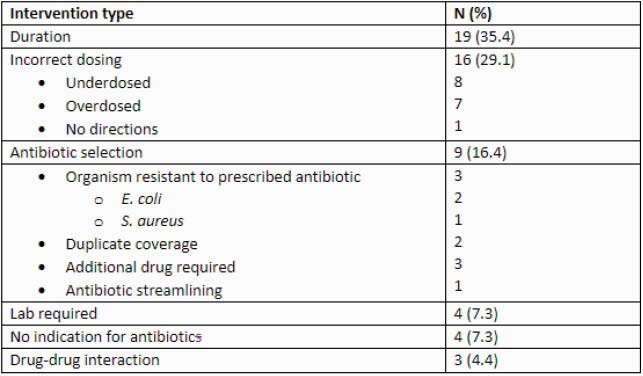

**Conclusion:**

Direct pharmacist review and intervention at discharge improved the prescribing of oral antibiotics within our institution during this pilot. Considering that this was conducted part-time in a subset of hospitalized patients during a limited time period, significant cost savings are possible with greater implementation.

**Disclosures:**

**Jason C. Gallagher, PharmD, FIDP, FCCP, FIDSA, BCPS**, **Astellas** (Consultant, Speaker’s Bureau)**Merck** (Consultant, Grant/Research Support, Speaker’s Bureau)**Qpex** (Consultant)**scPharmaceuticals** (Consultant)**Shionogi** (Consultant) **Jason C. Gallagher, PharmD, FIDP, FCCP, FIDSA, BCPS**, Astellas (Individual(s) Involved: Self): Speakers’ bureau; Merck (Individual(s) Involved: Self): Consultant, Grant/Research Support; Nabriva: Consultant; Qpex (Individual(s) Involved: Self): Consultant; Shionogi (Individual(s) Involved: Self): Consultant **Marissa Cavaretta, PharmD**, **Merck** (Grant/Research Support)

